# The political and scientific challenges in evaluating compulsory drug treatment centers in Southeast Asia

**DOI:** 10.1186/s12954-016-0130-1

**Published:** 2017-01-11

**Authors:** Thu Vuong, Nhu Nguyen, Giang Le, Marian Shanahan, Robert Ali, Alison Ritter

**Affiliations:** 1 The National Drug and Alcohol Research Centre/UNSW Australia, Sydney, Australia; 2 FHI360 Vietnam, Hanoi, Vietnam; 3 Hanoi Medical University, Hanoi, Vietnam; 4 University of Adelaide, Adelaide, Australia

**Keywords:** Compulsory treatment, Heroin use, Methadone treatment, Southeast Asia, Cost-effectiveness

## Abstract

**Background:**

In Vietnam, like many countries in Southeast Asia, the commonly used approach of center-based compulsory drug treatment (CCT) has been criticized on human rights ground. Meanwhile, community-based voluntary methadone maintenance treatment (MMT) has been implemented for nearly a decade with promising results. Reform-minded leaders have been seeking empirical evidence of the costs and effectiveness associated with these two main treatment modalities. Conducting evaluations of these treatments, especially where randomization is not ethical, presents challenges. The aim of this paper is to discuss political challenges and methodological issues when conducting cost-effectiveness studies within the context of a non-democratic Southeast Asian country.

**Methods:**

A retrospective analysis of the political and scientific challenges that were experienced in the study design, sample size determination, government approval and ethics approvals, participant recruitment, data collection, and determination of sources, and quantification of cost and effectiveness data was undertaken. As a consequence of the non-randomized design, analysis of patient characteristics for both treatment types was undertaken to identify the magnitude of baseline group differences. Concordance between self-reported heroin use and urine drug testing was undertaken to determine the reliability of self-report data in a politically challenging environment.

**Results:**

We demonstrate that conducting research around compulsory treatment in a non-democratic society is feasible, yet it is politically challenging and requires navigation between science and politics. We also demonstrate that engagement with the government decision makers in the research conception, implementation, and dissemination of the results increases the likelihood of research evidence being considered for change in a contentious drug policy area.

**Conclusions:**

Local empirical evidence on the comparative cost-effectiveness of CCT and MMT in a Southeast Asian setting is critical to consideration of more holistic, humane, and effective drug-dependence treatment approaches, but the garnering of such evidence is very challenging.

## Background

The problem of drug abuse and trafficking in Vietnam is long-standing, primarily due to its proximity to the “Golden Triangle”, the opium-producing region bordering Myanmar, Thailand, and Laos. The economic deregulation (known as *Doi Moi*) of Vietnam in 1986 enabled the introduction of market-oriented policies that facilitated the rapid growth of the economy and subsequent changes in lifestyles. One such change, especially in urban areas, was an increase in illicit drug use [1, 2]. The official number of people reported as dependent on illicit drugs increased quickly from 50,000 in 1995 to 150,000 in 2011 and 204,000 in 2015 [3]. Heroin has remained the primary drug of choice (84.7%) primarily via injection, followed by methamphetamine (5%), cannabis (1.3%) and pharmaceuticals (0.5%) [3].

Like many countries in Asia (11 countries), center-based compulsory treatment (CCT) is a common approach for resolving illicit drug use problems in Vietnam [4–7]. These countries (including Cambodia, China, Indonesia, Malaysia, Philippines, Thailand, and Vietnam) have endorsed policies that force people who use illicit drugs into some form of compulsory rehabilitation [8]. Largely based on a philosophy of “social reeducation”, compulsory rehabilitation gained momentum during the 1990s with the construction of large-scale centers in Malaysia, China, and Vietnam [9, 10]. This approach was imported by neighboring countries with an estimated 2 million people placed in compulsory centers in China and South East Asia in 2006 [11].

Compulsory rehabilitation centers have been criticized by the UN and human rights organizations for a variety of human rights abuses including involuntary indefinite detention, physical abuse, torture, and denial of or inadequate medical care [9, 12]. The first compulsory rehabilitation center in Vietnam was built in 1993 and by the end of 2015 there were 121 centers nation-wide with the placement of approximately 45,000 people at a time [3]. During the last two decades, the Vietnamese government has invested approximately US$47 million/year into this approach [13]. Yet, whether the centers are successful remains under debate [4, 14, 15] .People who use illicit drugs can be confined in the center for up to 2 years [4]. These centers are not part of the criminal justice system and their detainees have not necessarily been convicted of any crime [16].

The burden of illicit drug use on the health-care system is considerable. As a consequence of shifts in the route of heroin administration in most East and Southeast Asia countries from smoking to injecting and the associated sharing of injecting equipment, HIV prevalence among people who inject drugs dramatically rose. In Vietnam, injecting drug use accounts for two-thirds of all HIV cases [13]. The alarming twin epidemic of HIV and injection drug use attracted international donor agencies’ interest due to concerns about the potential for a more generalized epidemic. Considerable investment from international donors resulted in large-scale MMT programs as well as needle and syringe programs in many Asian countries, notably China, Malaysia and Vietnam [11]. In Vietnam, by March 2016, a total of 44,479 people had received MMT (27.3% of 163,000 dependent heroin users) [17]. However, the primary approach funded by the Vietnamese government for dealing with illicit drug use remains center-based compulsory rehabilitation (CCT).

Two recent events changed the landscape of drug policy in Vietnam in different but interrelated ways. The first was the release of a Human Rights Watch report into labor abuse of detainees in CCT centers in the South of Vietnam [18]. The second was the Vietnamese economy attaining middle-income status economy in 2010 [19], and hence no longer receiving international funding priority. Reform-minded Vietnamese policy makers were challenged by the reported limited success of the CCT centers approach [20] and the increasing number of dependent illicit drug users. In response, they reached out to international agencies for technical assistance to inform their investment mix. This included the provision of scientific evidence from economic evaluations of drug dependence treatment alternatives.

While considerable evidence of effectiveness [21, 22] and cost-effectiveness of MMT [23] exists, there was a lack of evidence regarding CCT, particularly in the East and Southeast Asian context. To date, only one study had been conducted in South East Asia. The study [24] conducted in Thailand evaluated the effectiveness of the Therapeutic Communities Program delivered in closed settings. While it found a large reduction in illicit drug use over 6 months, there was no control group and the highly significant contextual factors (the study was conducted from 2005 to 2007, only 2 years after the commencement of the Thailand war on drugs) may account for the results [25].

To address this lack of evidence an, evaluation of the relative effectiveness (as measured by heroin use, drug-free days, illegal behaviors, overdose, blood-borne virus risk behaviors and monthly drug spending) and cost-effectiveness (as measured by the cost to achieve “drug-free days” over three years, and the cost to achieve the proportion of people abstinence from illicit drug use) of CCT compared to MMT was undertaken in Vietnam (see [26]). The results of the cost-effectiveness study are reported elsewhere [26] where MMT was found to be superior to CCT in terms of both cost and effectiveness. This paper discusses the research challenges and solutions, provides the political context, and demonstrates the ability to undertake such research in a non-democratic society.

## Methods

A retrospective analysis of the political and scientific challenges experienced in the study design, study approval, data collection, ethics approvals, and analytic strategy was undertaken. Specifically, a thorough review of all versions of the study protocol and questionnaires, minutes of meetings held with the government of Hai Phong City, and all versions of the study timeline and participant recruitment timelines were undertaken. Due to the non-randomized design and to determine the magnitude of baseline differences, a comparison of participant characteristics for both treatment arms was undertaken. Concordance between self-reported heroin use and urine drug testing was examined to determine reliablity of self report research data in a politically challenging environment.

## Results

The results are presented chronologicaly, in terms of the issues we confronted, commencing with determining the most appropriate study design, followed by the study setting selection, the sample size, obtaining ethics approvals, strategizing participant recruitment, data collection methods, and sources and quantification of data (for both costs and outcomes/effectiveness).

### Designing the study

In an ideal world, a cost-effectiveness study would entail a randomized design, where participants are randomly allocated to one of two groups: CCT and MMT. However, it was not ethical to randomize people to CCT - an intervention which violates human rights. It would also not have been practical to randomize participants inasmuch as those randomized to CCT would most likely withdraw from the study. In this context, a quasi-experimental design was the only feasible alternative but this posed further challenges in that there could potentially be significant differences between the two groups because of the lack of random assignment.

Table 1 provides the participants’ demographic characteristics by treatment group. One hundred percent of the participants in both groups were male because all three CCT centers involved in this study were for male illicit drug users. The CCT group was significantly younger (mean = 33.26 vs 37.32 years), more likely to be single (51.69 vs 43.49%), more likely to be employed (76.45 vs 66.67%) and had higher legal monthly income (3.00 million VND versus 1.50 million VND). There were no statistically significant differences between the two groups in education with 39.90% of CCT participants and 46.09% of MMT participants having finished high school (12 years of education) at minimum.

**Table 1:** Participants’ demographic characteristics by treatment group

	CCT (*n* = 208)	MMT (*n* = 384)	Test result and *P* value
Male (%)	100	100	n/a
Mean age (SD)	33.26 (7.60)	37.32(8.23)	*t*(590) = -5.624; *p* < 0.001
Marital status (%)			
Single	51.69	43.49	*X* ^2^(1) = 4.16; *P* = 0.04
Married, divorced, separated or widow	48.31	56.51	
Employment (%)			
Unemployed	23.56	33.33	*X* ^2^(2) = 11.46; *P* = 0.003
Full-time	17.31	21.35	
Part-time (casual work)	59.14	45.32	
Average legal monthly income (median and range)	3.00 million (0.80–15.00 million)	1.50 million (0–35.00 million)	U = 28,617; *p* < 0.001
Education (%)			
≥High school	39.90	46.09	*X* ^2^(1) = 1.95; *p* = 0.16
<High school (year 12)	60.10	53.91	

Table 2 summarizes participants’ drug use history and behavioral characteristics by treatment group. Both groups started to use drugs in their early 20s. The CCT group had a higher proportion commencing with heroin as the first drug used (85.58 vs 80.73%) compared to the MMT group but their median daily use frequency was lower (three times a day vs four times a day) and they had been using drugs for fewer years (11.01 vs 13.17 years). Three months prior to commencing either MMT or CCT, the proportion of people who used heroin daily was not statistically different between two groups (CCT = 97.50%; MMT = 99.96%). From the commencement of drug use until treatment entry, the CCT group spent less money on drugs on a monthly basis (4.55 million dongs (~US$213) vs 6.05 million dongs (~US$290)), which is consistent with using less frequently.

**Table 2:** Participants’ drug use history and behavioral characteristics by treatment group

	CCT (*n* = 208)	MMT (*n* = 384)	Test result and *P* value
Mean age of first intoxication (SD)	22.35 (5.79)	23.22 (6.42)	*t*(590) = -1.68; *p* = 0.09
% drugs of first intoxication			
Heroin	85.58	80.73	*X* ^2^(2) = 7.26; *p* = 0.03
Opium	10.58	16.41	
Other drugs	4.81	2.34	
Mean number of years used (SD)	11.01 (6.22)	13.17 (4.63)	*t*(590) = -5.08; *p* < 0.001
Ever reported daily heroin use (%)	97.54	99.96	*X* ^2^(1) = 3.57; *p* = 0.06
Median heroin use frequency *of those who used every day* (range)	3 (0–20)	4 (0–18)	*U* = 64,077; *p* < 0.001
Median monthly drug spending (range)	4.55 mil VND (0–84 mil)	6.20 mil VND (0–225 mil)	*U* = 60,171; *p* < 0.001
% heroin injection ever	66.43	82.55	*X* ^2^(1) = 30.79; *p* < 0.001
% poly drug use ever^a^	50.48	28.91	*X* ^2^(1) = 29.99; *p* < 0.001
Mean number of drug classes used (SD) *of those who were poly users*	3 (1.25)	2 (0.82)	*t*(214) = 4.69; *p* < 0.001
% ever sought drug dependence treatment	80.77	96.61	*X* ^2^(1) = 55.14; *p* < 0.001
Median number of treatment episodes (range)^b^	2 (1–50)	5 (1–40)	*U* = 49,581; *p* < 0.001
% ever been to CCT centers	37.98	49.74	*X* ^2^(1) = 8.46; *p* = 0.004
% ever home detoxed	66.70	84.40	*X* ^2^(1) = 27.67; *p* < 0.001
% ever committed illegal behaviors	35.20	30.30	*X* ^2^(1) = 2.35; *p* = 0.13
% ever been to prison	16.35	26.30	*X* ^2^(1) = 7.59; *p* = 0.006
% overdose incident ever	18.75	12.10	*X* ^2^(1) = 3.49; *p* = 0.06

^a^Methamphetamine second drug of choice 28.30% for CCT and 11.60% for MMT

^b^For those who ever sought treatment

A smaller proportion of the CCT group reported having ever injected heroin (66.43 vs 82.55%). However, the CCT group were more likely to be poly drug users (50.48 vs 28.91%), with 28.30% of CCT participants and 11.60% of MMT participants also using methamphetamine. Among those who were poly drug users, the CCT group used a higher number of drug classes (3 vs 2). In terms of drug dependence treatment history, CCT participants were less likely to have sought treatment (78.77 vs 96.61%) and, for those who did, the median number of treatment episodes was fewer (2 vs 5). CCT participants were less likely to have previously been in CCT centers (37.98 vs 49.74%) and less likely to have undergone home-based detoxification (66.70 vs 84.40%). Even though there was no statistically significant differences in the proportions of people who reported ever committing illegal behaviors (CCT = 35.20% and MMT = 30.30%), a significantly higher proportion of MMT participants had ever been to prison (26.30 vs 16.35%). There was no statistical difference between the two groups (CCT = 18.50% and MMT = 12.10%) in the proportion of participants who had experienced a drug overdose.

Because this study was an observational, non-randomized study, baseline characteristic differences between groups were not unexpected. Overall, CCT participants appeared to be less dependent on illicit drugs compared to MMT participants at baseline. This potentially biased the study in favour of CCT over MMT. Statistical techniques that accommodated baseline differences were essential. This entailed the use of mixed effects regression models with covariance analysis adjustments (see details in [26]).

The second design consideration was the time and available resources for the study. Again, in an ideal world, both groups would be followed prospectively, with similar timeframes (such as data collection at admission, and then at subsequent follow-up points). However, a comprehensive study of MMT had already been undertaken in Vietnam, which provided a potential sampling frame for the MMT arm of this study [27]. Commencing a new MMT cohort would require nearly double the funding and two additional years of research implementation.

The final study design built on the absence of randomization and the investment that had been made in the previous MMT study. The result was a combined retrospective and prospective quasi-experimental cohort study with five time-points; baseline (B), 2 years after treatment commencement (T1), then 3 months (T2), 6 months (T3) and 12 months (T4) prospective follow-up. The MMT participant group was drawn from the 384 MMT participants from the previous study at their 24-month post-treatment interview. They were then invited to participate in the current study. The CCT group was recruited at point of exit from the CCT centers (that is, after 2 years in the program) which resulted in a notional equalization between the two groups in terms of time since intervention commencement. The results are available at [26].

### Study setting

An informed decision needed to be made when determining potential research settings. In an ideal world, the study sites would be randomly selected, however pragmatic practical considerations ensuring feasibility meant that a single city was selected: Hai Phong City. Hai Phong City is one of only two cities in Vietnam where both CCT and MMT programs were well-established with standardized policies and procedures. Second, it was the only city where the political leadership was willing to work with international organizations to review their drug dependence treatment system so as to improve effectiveness and efficiency. Third, FHI360, a US-based non-governmental organization and one of the three institutions involved in facilitating this research, had already developed a long-term relationship with the Hai Phong government leaders through the funding and implementation of a comprehensive HIV prevention and drug dependence treatment program including MMT.

The ultimate goal of conducting this research was to generate evidence that would support a dialogue for drug policy change. Therefore, engaging policy-makers and government leaders of Hai Phong City in the entire process of conceptualization, conduct, interpretation, and dissemination of the research was critical. As such, a series of consultations between the research team and the Hai Phong city government leaders and relevant departments took place in early 2012. Despite the long-term relationship and trust between FHI360 and Hai Phong City government, getting political approval for this study was not an easy task; it took nearly 2 years to obtain approval. The political support of the local government was critical for permission to interview CCT participants and for access to CCT centers’ costing data. Most importantly, the political leadership expressed a commitment to review the findings of the study, engage in discussions around those findings and consider possible change to resource allocation taking the study findings into account.

CCT has existed in Hai Phong City (a coastal city in Northern Vietnam) since 1991. Significant investment has been made by the city government to the three CCT centers with a combined capacity of approximately 2500 people (out of an estimated 8000 people dependent on illicit drugs). In the community, if someone is identified as using illicit drugs, they will be encouraged to undergo community-based detoxification treatment. If they fail to stop using drugs after several attempts, they are sent to a CCT center for a period of two years. People can also be arrested during ‘special cleaning up’ occasions and if their urine drug test is positive they may be sent to the CCT centers. In the centers, the “rehabilitation” strategies focus on moral teaching, basic health care services, “cold turkey” detoxification, and forced labor work [28].

MMT was first piloted in Hai Phong City (three clinics) and Ho Chi Minh City in 2008 and from 2011 was endorsed by the government for rapid expansion nation-wide. In Vietnam, MMT requires daily supervised dosing by clinic staff. As part of the treatment protocol, random opiate urine drug testing occurs on a monthly basis. If the patient continues to use opiates while in MMT treatment, he/she is provided with relapse prevention counseling services.

### Sample size

Determining the appropriate sample size for this study needed to address three aspects: 1) the lack of previous research on CCT; 2) the non-randomized design and; 3) potential loss to follow-up. The primary outcome measure used in the power analysis was the estimated proportion of people to be heroin abstinent at the final time-point (T4). The calculation used an alpha of 0.05, beta of 0.20 (power 80%) and an estimated proportion of successful treatment (heroin-free) of 20% at 12 months after being released from CCT centers (T4).This estimate had been reported by the governments of many provinces/cities in Vietnam, using administrative data. To be conservative, we assumed that the CCT in Hai Phong City was more effective compared to the rest of Vietnam with a success rate of 42% at 12 months following release. This assumption was based on the higher level of investment in facilities and human resources. For MMT treatment, the 2009 MMT cohort study reported 85.4% of the MMT participants who remained in treatment were heroin abstinent at 12 months. The power analysis assumed that the proportion of MMT participants who were free of illicit drug use would be 62.4% if the data analysis included both missing and censored data. These assumptions determined a minimum sample size of 136 per group which was increased to 186 given an estimated 30% loss to follow-up. Due to the non-randomized study design, baseline group differences were expected and therefore the final sample size of 208 (for each group) was determined.

Additional consideration was given to the appropriate selection of the MMT sample: a choice between the 384 MMT participants at T1 or only the 314 who consented to participate in the follow-up time periods (T2). A decision was made to select the 384 people who were interviewed at 2-year post-treatment as the starting sample. This strategy aimed to equalize the starting samples of both the CCT and MMT groups to the number at the end of 2-year post treatment. This potentially biases against MMT, inasmuch as there are no follow-up data for the 70 MMT participants (384–314) who were unable to be contacted. Their outcome data were then imputed using a worst case scenario assumption.

### Ethics approvals

Ethics approvals were required and granted by three institutions: the University of New South Wales (#HC12259, dated 1 August 2012), Hanoi Medical University (#106/HMU IRB dated 2 October 2012), and FHI360 Headquarters in NC, the USA ([408418-1]#10389, dated 13 December 2012). It took between three and five months for each approval. Each ethics application required that all participant information, consent forms and questionnaires be provided in both English and Vietnamese. This required fluency in both English and Vietnamese, and competency in the technical content. In addition, the research proposal, the Participant Information Statement, Consent Form and questionnaires were submitted to the Local Review Committee which comprised the Hai Phong City local Government agencies (#4219/UBND-VX dated 17 June 2013). The Participant Information Statement/Consent Form included one section on consent from the participants for their data to be published.

The interactions with the Hai Phong City local Government agencies were critical for two reasons. Firstly, the Hai Phong city government was sensitive about endorsing an international organization conducting research involving CCT centers, particularly when this approach had been criticized openly both at national and international fora by human rights organizations. Without the opportunity to review the study documents, the Hai Phong city government would not have been confident to endorse the research. Secondly, engagement of the policy-makers who could potentially use the study results to formulate actions increases the likelihood of those findings being applied. There were some concerns that these interactions around the study formulation would jeopardize the objectivity of the study. Therefore, one strategy employed was to facilitate the establishment of a Local Review Committee such that it comprised a full range of stakeholders including the health department, police department and labor and social affairs departments. Clear roles, responsibilities and accountabilities were defined whereby the researchers, not the committee, were responsible for the final product. This arrangement of roles and responsibilities enabled the government staff to function in their role while contributing to the quality of the final product.

Due to the concern about the potential reporting of human rights violations as well as behaviors of the CCT participants while in the CCT centers, the Local Review Committee requested the research team remove all questions pertaining to drug use behaviors as well as other activities of the CCT participants while in CCT centers. This was the only substantial request. This had a significant impact on the study because data regarding drug use behaviors during the two-year period of CCT rehabilitation were not collected. This requirement necessitated the use of proxy data (discussed below). While the use of proxy data for estimating drug use behaviors is less desirable, the trade-off was needed for the study to proceed and for the continued support of the local government. This was not only for the implementation of the study but also for the support for dissemination of the study findings.

### Recruitment challenges

All CCT participants who were released from the three CCT centers during January-November 2013 were invited to take part in the study. In order to recruit the 208 CCT participants, 550 invitation letters were sent to eligible CCT participants by the CCT center staff. As it would be unethical for the researchers to be aware of the names of the CCT participants without their agreement, the researchers provided the invitation letters to the CCT center staff to distribute. Approximately 30% of the letters were returned due to unidentifiable postal address. Therefore, two additional recruitment strategies were introduced: (1) referral by CCT-released participants who had already enrolled in the study; and (2) referral through the peer educators’ network. Specifically, at the end of each interview with a CCT-released participant, the interviewer provided three copies of the information leaflet and asked them to assist by providing the leaflet to people who they believed would be eligible for the study. The leaflet indicated that eligible participants were required to show the official certificate of treatment completion. The research field investigator also contacted the peer education program supported by FHI360 to talk to 16 peer educators and ask them to assist by delivering the information leaflet to people they identified as eligible for the study. The proportion of enrolled participants as a result of being referred by these additional means was 29.8% (62 out of 208).

The final sample for the CCT arm at study commencement was 208 (54% of the CCT exits). The non-participation rate was largely due to an inability to contact potential participants. The sample size required for sufficient statistical power was achieved for CCT participants and exceeded for MMT participants (*n* = 384). This demonstrated the feasibility of recruiting voluntary participation in a drug treatment study, including vulnerable groups who had been involuntarily detained for 2 years even though they may rightly have been suspicious of any government-associated research.

The intent was to collect data from both groups across five time-points (baseline, two years after treatment commencement, then 3, 6, and 12 months after the initial two years). A substantial delay in obtaining political endorsement from the Hai Phong City Government meant there were variations between the original planned timeline and the actual recruitment and follow-up interviews. Specifically, the time between the first follow-up interview (T1) and the second follow-up interview (T2) for the MMT group was 19 months (the time between the end of the previous MMT study and the beginning of the current study) while it was 3 months for the CCT group. For the CCT group, there was an average of 4 months between the time of release from the CCT centers and the T1 interview. Combined, the total time between baseline and the final follow-up (T4) for the MMT group was approximately 5 years while it was 3.5 years for CCT group.

This non-equivalency of time was managed by including time as a covariate in the data analysis, allowing the mixed effects regression modelling to both account for time as a covariate, and assess the statistical significance of between group differences in relation to time trends. This aimed to ensure that the differences in time would not create a bias in the effectiveness results comparing two groups.

Despite the marginalized nature of the study participants, the research team was able to follow-up 80% of the CCT participants and 78% of the MMT participants at the end of the study (T4: 12 months post two years treatment). The follow-up rate at 3 (T2) and 6 (T3) months for the CCT group was 88 and 83%, respectively while the follow-up rate at T2 and T3 for the MTT group was 82 and 80%, respectively. Achieving adequate follow-up rates with people who are dependent on drugs and alcohol, particularly participants who are not in treatment is challenging. Reviews of longitudinal research designs have identified a range of strategies for maintaining contact with participants and obtaining high response rates of approximately 70–80% [29]. One of the strategies involves the collection of detailed locator information at the start of the study. In this study, at the baseline interview standardized locator information was collected including current contact details, names and contact details of family members or next of kin. This was reviewed at each follow-up interview. To ensure systematic implementation of follow-up procedures across the ten field researchers and to increase the likelihood of participant follow-up, a “Participant Tracking Protocol” (see Table 3) was developed with inputs and consensus from all the ten local researchers. This participatory approach was used to increase the sense of ownership thereby increasing the likelihood of them implementing the protocol.

**Table 3:** Participant tracking protocol

This protocol was developed as a guidance for the study interviewers in their tracking of the study participant to improve successful follow-up of the study participants. 1. One month after last interview, call the participant to ensure that their phone number is still contactable, and remind them of the next approximate date for the next interview. o Call the participants: 3 times/3 days/3 different times in the day (morning, afternoon, evening). o The first call if the phone rings but not being picked up, send a text message (introducing name, from Hai Phong Medical University) to inform will call again. o If the above don’t work, call next of kin. 2. Call the participant’s family members or friend that are listed in the consent form the participant provided (if any because some do not want to provide): for each locator (family member or friend) follow the same procedures below: o Call next of kin: 3 times/3 days/3 different times in the day o If the phone rings but not being picked up, send a text message (introducing name, from Hai Phong Medical University) to inform will call again. → If (1) and (2) don’t work, move to step 3 and 4 concurrently. 2. Interviewer check the excel administrative list of participants, use excel search tool to look for participants who have somewhat similar home addresses (they live near each other), call 1 or 2 participants whose address is near to ask them to give a message. 3. Use the address they provided in the consent form, send them a reminder note (using sample/format provided). 4. f they do not return the call after 4 days (from the send date of the reminder note), use the address they provided to look for their home to see if they still live there. 5. Two months after the interview, repeat the steps from 1 to 5; 6. If still not successful, wait until 7 days before the scheduled next interview, repeat the steps from 1 to 5; 7. If still not successful, skip this interview (consider missing of 1 wave); 8. Repeat steps from 1 to 5 for the future waves;	

Note:

Each interviewer has been provided with a tracking book with tables laid out, please remember to take notes of all information related to dates of phone calls, dates of sending text messages, dates of sending letters, dates of home visits and other related information that are helpful to assist successful tracking of participants

### Data collection challenges

The selection and design of the data collection instruments was limited by the previous MMT study (the source of the baseline data for the MMT group). The structured questionnaire was administered by trained interviewers, and asked about the participants’ socio-demographic information, drug use behaviors, drug-use related illegal behaviors, blood-borne virus risk behaviors and overdose experience, for their lifetime, for the preceding 3 months prior to index treatment, at 2 years after treatment commencement (from the release date to the T1 interview date for CCT participants). At the 3-month (T2), 6-month (T3) and 12-month (T4) interviews, the same questions were asked for the period of the preceding 3 months. At the end of each interview with CCT participant, a urine drug sample was provided by the participant for opioid drug screening. The urine drug test results of MMT participants were retrieved from their patient records at the MMT clinics. As per treatment protocol, opioid urine drug testing is carried out at random for MMT patients on a monthly basis. The urine drug test results were used to verify the validity of their self-reported heroin use. For both CCT and MMT participants, the Alere© MOP One Step Morphine Test was used for urine drug screening. The test utilizes a monoclonal antibody to selectively detect elevated levels of morphine (the metabolite of heroin) at a cut-off concentration of 300 ng/mL.

Contrasting data from urine tests for heroin with self-report of heroin use yielded four possible scenarios: A) negative urine and negative self-report; B) positive urine and negative self-report; C) negative urine and positive self-report; and D) positive urine and positive self-report. In order to calculate the percentage of concordance between self-report and urine screen results, the percentage of scenario A and scenario D were added together. The percent (%) agreement was calculated based on the number of valid cases, i.e., cases that were not lost-to-follow-up at the relevant time-point.

For CCT participants, the overall concordance between self-report and urine screen ranged from 80–86% from T1 to T4 (Table 4) (baseline drug screening results were not available for CCT participants because the first interviews were conducted at T1 and self-report data on baseline behaviors were collected retrospectively). For MMT participants, the overall concordance between self-report and urine screen ranged from 82–88% from T1 to T4 (Table 5).

**Table 4:** Agreement of self-reported heroin use and urine drug screens: CCT participants

			Urine drug screen results		
Self-report	Negative	Time-points	Negative	Positive	Totals
			A	B	
		Baseline	n/a	n/a	n/a
		T1	60/208 (29%)	27/208 (13%)	87/208 (42%)
		T2	57/182 (31%)	17/182 (10%)	74/182 (41%)
		T3	46/173 (27%)	20/173 (11%)	66/173 (38%)
		T4	54/166 (33%)	19/166 (11%)	73/166 (44%)
	Positive		C	D	
		Baseline	n/a	n/a	n/a
		T1	8/208 (4%)	113/208 (54%)	121/208 (58%)
		T2	7/182 (4%)	101/182 (55%)	108/182 (59%)
		T3	15/173 (9%)	92/173 (53%)	107/173 (62%)
		T4	9/166 (5%)	84/166 (51%)	93/166 (56%)

*The percentage (%) of agreement was calculated based on the number of valid cases, i.e., cases that were not lost-to-follow-up at the corresponding time-point

**The concordance rates were calculated by dividing the number of cases with responses matched by the number of valid cases

Concordance of agreement = A + D:

T1 = 29% + 54% = 83%

T2 = 31% + 55% = 86%

T3 = 27% + 53% = 80%

T4 = 33% + 51% = 84%

**Table 5:** Agreement of self-reported heroin use and urine drug screens: MMT participants

			Urine drug screen results		
Self-report	Negative	Time-points	Negative	Positive	Totals
			A	B	
		Baseline	2/384 (1%)	0/384 (0%)	2/384 (1%)
		T1	294/384 (77%)	46/384 (12%)	340/384 (89%)
		T2	251/314 (80%)	32/314 (10%)	283/314 (90%)
		T3	258/302 (85%)	26/302 (9%)	284/302 (94%)
		T4	260/298 (87%)	19/298 (7%)	279/298 (94%)
	Positive		C	D	
		Baseline	13/384 (3%)	369/384 (96%)	382/384 (99%)
		T1	23/384 (6%)	21/384 (5%)	44/384 (11%)
		T2	25/314 (8%)	6/314 (2%)	31/314 (10%)
		T3	12/302 (4%)	6/302 (2%)	18/302 (6%)
		T4	16/298 (5%)	3/298 (1%)	19/298 (6%)

*The percentage (%) of agreement was calculated based on the number of valid cases, i.e., cases that were not lost-to-follow-up at the corresponding time-point

**The concordance rates were calculated by dividing the number of cases with responses matched by the number of valid cases

Concordance of agreement = A + D:

Baseline = 1% + 96% = 97%

T1 = 77% + 5% = 82%

T2 = 80% + 2% = 82%

T3 = 85% + 2% = 87%

T4 = 87% + 1% = 88%

Overall, the concordance between self-reported heroin use and results from urine drug screens was high for both groups and suggested that the participants of both groups reported their heroin use accurately.

As noted above, it was not possible to collect data on drug-using behaviors of the CCT participants while being in CCT centers. For this study, the primary outcome was the number of drug-free days over 3 years (two years in CCT centers plus 1 year in the community for CCT participants). Therefore, the number of drug-free days for the two year stays in CCT centers needed to be estimated. Proxy data of drug from behaviors reported among prison populations was identified and used because the conditions under which CCT centers operate are similar to the conditions under which international prisons operate, such as security, discipline, no-tolerance of illicit drug use policy. As such, the proxy data were derived from the reported prevalence and frequency of illicit drug use among prison populations in other countries [30–35]. Specifically, the six referenced studies provided evidence that the illicit drug use prevalence among drug-using prison inmates ranged from 31 to 74% and the frequency of use ranged from 1 to 8 days in the last 30 days. Derivation of a number that could be representative of these studies was impossible. Therefore, a decision was made to use a generalized average of 26 drug-free days out of 30 for imputation (for the 67% of the CCT participants who reported drug use at T1) for their 2-year stay in the CCT centers. The remaining 33% of the CCT participants did not report illicit drug use at T1, hence were assumed to be less likely to have used illicit drugs while in the CCT. It was believed that this assumption was reasonable because drug use occurrence has been reported in the Vietnam CCTs [36, 37].

Even though quality-adjusted life years (QALYs) is a critical measure of effectiveness in economic evaluations, it was not possible to use QALYs as an outcome measure for this study. The reason was because by guideline, the tools used to measure quality of life (EQ-5D, SF-6D, HUI) apply to a respondent’s immediate situation. They are not intended to be used to summarize the health status recall over the preceding weeks, months, or in our case, years. The design of this study required recall of data up to 24 months (for CCT participants). Therefore, estimating the change in QALYs of the participants was not technically possible.

### Sources and quantification of effectiveness data

Table 6 provides information on the six outcome measures for the comparison of effectiveness, with details on where each of the outcome measures was sourced from and how each of them was quantified into values that were later used for the data analysis.

**Table 6:** Six outcomes measures for comparison of effectiveness: sources and quantification

Outcome measures	How the outcome measures were sourced and quantified
Comparison of effectiveness	
Heroin use	Based on self-reported heroin use at each of the five time-points. The question was ‘During the last 3 months, did you use heroin?’ The answer was ‘Yes’ or ‘No’ (dichotomous outcome variable created in the data set with Yes = 1 and No = 0). Results of urine drug screening were used to validate self-reported heroin use.
Drug-free days (in the preceding 30 days)	The question asked was ‘During the previous 30 days, on average, how many days did you NOT use any illicit drugs?’ The value to this question formed the basis for a continuous outcome variable with the range from 0 to 30 (drug-free days).
Drug-use related illegal behaviors	A list of 6 questions about drug-use related illegal behaviors was asked; each focused on a type of illegal behavior: a) Using force to get money from other people; b) Fraud; c) Theft; d) Robbery; e) Illicit drug dealing; and f) Assault, physical flight to get money to buy illicit drugs. A dichotomous variable (Yes/No) was created for this outcome measure with all illegal behaviors combined.
Drug-used related BBV risk behaviors	The BBV-TRAQ-SV [46] was used to create data for this outcome measure. Answers to all questions were counted and a single summary score was calculated.
Overdose incident	The question was ‘Did you experience any overdose incident during the previous 3 months?’ A dichotomous outcome variable was created with Yes = 1 and No = 0.
Monthly drug spending	Answers to 4 questions were used to calculate the value for this outcome variable: ‘The number of days using illicit drugs monthly?’ ‘List of the three drugs that you used the most frequently during the last 3 months?’ ‘For each of the three drugs, how much money did you spend for one time of use?’ ‘What was the frequency of use for each of the 3 drugs?’ The following formula was used to calculate the amount of monthly drug spending for each drug: MonthlySpending = (NumberOfDaysUse*DrugDailyFrequency*AmountSpent) + (DrugWeeklyFrequency*4 (weeks)*AmountSpent) + (DrugMonthlyFrequency*AmountSpent) The amount of monthly spending for all three drugs were added together and the value formed a continuous outcome variable.

Table 7 provides information on two outcome measures for the comparison of cost-effectiveness with details on how each of them was quantified into values that were used for the data analysis.

**Table 7:** Two outcomes measures for comparison of cost-effectiveness: sources and quantification

Outcome measures	How the outcome measures were sourced and quantified
Comparison of cost-effectiveness	
Heroin abstinence	The mechanics of the transfer of the results from the effectiveness analysis over to the CEA for ‘heroin abstinence’ outcome followed three steps: Step 1: The mixed effects regression analysis conducted on ‘heroin use’ produced individual-specific predicted values pertaining to the probability of ‘heroin use’ at each of the five time-points. Step 2: The individual-specific predicted values of the probability of ‘heroin use’ at the final time-point was selected, and then manually reversed to become the probability of ‘heroin abstinence’. For example, if the probability of ‘heroin use’ of one participant was 0.78, the probability of ‘heroin abstinence’ of this participant would be 1–0.78 = 0.22. Step 3: These individual-specific predicted values of the probability of ‘heroin abstinence’ were then used for estimating the ICER together with the cost data.
Drug-free days (over 36 months)	The mechanics of the transfer of the results from the effectiveness analysis over to the CEA for DFDs followed three steps: Step 1: The mixed effects regression analysis conducted on DFDs (in the preceding 30 days)’ produced the individual-specific predicted values for each of the five time-points. Step 2: Using these individual-specific predicted values of all five time-points to calculate the aggregated value over 36 months, using the formula described above. Step 3: The individual-specific aggregated values for the DFDs (over 36 months)’ were then used for estimating the ICER together with the cost data.

### Sources and quantification of cost data

The perspective taken for the cost-effectiveness analysis (CEA) was that of the treatment sector. The average program costs (total program costs for each MMT clinic and CCT center divided by the number of participants) were added to the individual-specific participant costs to derive an individual-specific total cost. Although both participant and program costs were included, it was not a full societal perspective as it did not include other health care or criminal justice costs. Program costs included all costs required for the functioning of a CCT center or a MMT clinic, except for the cost of land. Participant personal costs included costs paid by the participants and the opportunity costs (travel costs and loss of productivity) of attending treatment. Loss of productivity was calculated using the reported times for both groups and the individual-specific monthly wage. MMT program cost data came from: (1) a previous MMT cost study that provided 2009 program costs [38]; and (2) a MMT program 2013 expenditure report provided by the Hai Phong City Department of Health; and (3) individual-specific methadone doses from which the individual-specific methadone syrup cost was calculated. CCT program costs were 2010, 2011, and 2012 financial data, provided by three CCT centers through a modified DATCAP questionnaire [39]. A detailed description of the cost components and the sources of data are presented in Table 8. The average annual MMT and CCT program costs were averaged across the different years, after adjustment for inflation using the 2013 general Consumer Price Index for Vietnamese Dong (VND) [40] as well as adjusting for participant drop-out at each time-point.

**Table 8:** Cost components of MMT and CCT

Program costs	Participant costs
For MMT:	
*Recurrent costs:* variable costs (salary for staff, methadone, urine tests and consumables), and fixed costs (site operation and maintenance, medication import and distribution) *Capital costs* ^a^ *:* Buildings and equipment/vehicles (10% depreciated value over 10 years) *Data sources:* + Secondary data of the 2009 costing study +2013 expenditure data provided by Hai Phong City Department of Health	*Cost of treatment:* zero because the treatment was free to the participants *Opportunity costs:* Costs of travel time to MMT clinic on a daily basis including waiting time Cost of petrol for travel to the MMT clinic on a daily basis *Data sources:* + Data collected through the modified DATCAP questionnaire – Outpatient version, used once for the baseline interview
For CCT:	
*Recurrent costs:* variable costs (salary for staff, medicines, biological tests and consumables), and fixed costs (center operation and maintenance) *Capital costs:* ^a^ Buildings and equipment/vehicles (10% depreciated value over 10 years) *Data sources:* + Data collected through the structured questionnaire that was based on the framework of the DATCAP questionnaire–Program version	*Cost of treatment* during the 2 years in CCT centers: + Rehabilitation costs paid by participants to the centers + Costs of food and other supplies provided by families to the participants on a monthly basis *Opportunity costs:* Loss of income due to 2-year placement in CCT centers (based on self-report monthly income and employment status at 3-month prior to CCT placement) *Data sources:* + Questions asked once, included in the outcome questionnaire for the baseline interview

^a^Value of MMT clinics land and CCT centers land was not collected (to be discussed in the Discussion section)

For this study, it was not possible to estimate the land cost of CCT centers which would be substantial given that CCT centers use a large area of agricultural or forestry land. Inclusion of land costs in the study required complicated assessment of land value (with specific procedures required by the Vietnam Ministry of Finance) that was not feasible within the study framework and resources. In addition, the governments accounting at the time of the study (and also at the present time) excluded the land value from the costs of public services. The study, therefore, excluded the land cost from the data collection. Similarly the value of production from this land was not included. Data on land and facilities of MMT clinics were available but were not used because land costs of CCT centers were not available.

Program and participant personal costs were calculated for one year before combining these two components to derive 1-year total average cost. Because the time horizon of the cost-effectiveness analysis was 3 years, the 3-year average total costs were based on the 1-year average total costs and the individual-specific number of years in treatment. Here, we faced a challenge pertaining to the time in-equivalence of the two treatment modalities, in that the MMT treatment was an on-going intervention while the CCT rehabilitation was time-limited for 2 years, with no costs incurred in the third year. To resolve this we used a pragmatic strategy. For MMT, the average three-year program costs were calculated by multiplying the average annual program costs by three (with adjustment for drop-out). For CCT, the average 3-year program costs were calculated by multiplying the average annual program costs by two. Every CCT participant, by law, completed the 2-year CCT compulsory treatment therefore adjustment for drop-out was not required.

## Discussion

### Science and politics in study design and implementation

We have demonstrated that conducting research around compulsory treatment in a non-democratic society is feasible. However, the study confronted a number of ethical, logistical, methodological, and political challenges, which affected several aspects of the study.

In 2011, Human Rights Watch criticized the Vietnamese government and many international aid organizations for supporting the CCT center system in Vietnam and claimed that the system “infringes on human rights standards”. The criticism was voiced through the release of a report titled “The Rehab Archipelago: Forced Labor and Other Abuses in Drug Detention Centers in Southern Vietnam”, providing evidence on inhumane treatment and labour abuse of people who use drugs in many CCT centers in the south of Vietnam [18]. Being publicly criticized in various international fora, international aid organizations were forced to devise strategies to disengage from supporting the CCT centers [28]. Similar to other Southeast Asian governments who have also been criticized on this issue, the Vietnamese government exhibited a diplomatic attitude in handling human rights criticisms. Yet, they also expressed their confusion about why compulsory treatment in Vietnam was criticized when it was seen as acceptable in developed countries and when UNODC in their endorsed principles of treatment claimed that “compulsory treatment could be effective” [41]. Furthermore the INCB had called on Vietnam to “reinforce and support existing facilities” [42]. As such, the debates around the ethics of compulsory centers appear to see no resolution in sight [14, 15, 43].

Perceptions about the impact of the criticisms by human rights organizations towards the Vietnamese government (and other Southeast Asian governments more broadly) pertaining to the CCT centers approach have varied. On the one hand, the human rights arguments have been perceived to be confusing, unhelpful and at times counter-productive because different societies and cultures define human rights differently and Western interpretations of human rights are viewed by Asian leaders as unpalatable [44]. On the other hand the efforts by human rights organizations have been perceived as achieving their goals to a substantial extent. While there may have been no direct impact on the Vietnamese government, they have helped raise awareness among international aid organizations, including international donors and UN agencies. It has demanded a rethink and more sophisticated responses by international aid organizations. Subsequent actions by international agencies took various forms: a) engaging in a more direct dialogue with the government on shifting away from compulsory centers approach (conversations which were often avoided in the past); and b) withdrawing funding from CCT centers because any support of the system was considered as legitimizing its continuance. Five years on from the release of the Human Right Watch’s report, the number of CCT centers in Vietnam had not changed. But the Vietnamese government introduced several legal documents that made it harder to send people who use drugs to CCT centers. As a consequence, many CCT centers have much fewer residents than the designed capacity.

In the meantime, a more effective approach over and above human rights arguments is the economic argument (i.e., scientific evidence from an economic evaluation), which engaged the Vietnamese government with the aim of practical change. An economic argument is believed to be more straightforward, less aggressive and therefore more likely to be considered by Vietnamese leaders. However, at the inception of this study, more than once, the Vietnamese research team (more specifically TV) was challenged by various staffers of international aid organizations in Vietnam about what strategies should be in place if the results of such an economic evaluation showed that CCT was more effective or more cost-effective than MMT. As such, there were two levels of risk in conducting this study: the risk involved with its implementation and the risk pertaining to what should be done if unexpected results were realized.

Notwithstanding the risk of the possibility of unexpected results, this study faced challenges in getting political endorsement for its implementation in the territory of Hai Phong City, especially when the approval was sought 1 year after the release of the Human Rights Watch report. It took 2 years for the political endorsement to be finally granted (in February 2012). However, in July 2012 there was a change in the leadership of the Department of Labor, Invalids and Social Affairs (DOLISA), the department in charge of the CCT centers. This required a new approval, which was not granted until June 2013. The new approval was possible at the cost of a series of negotiations and compromises and the cost of a substantial delay of implementation (approximately 12 months). The compromises included a) removal of questions on drug-using behaviors of CCT participants while being in CCT centers and b) constraints on location where interviews with CCT participants could be conducted. At one point it was believed that the new approval would not be possible. The primary reason for the uncertainty was because the Hai Phong City government was concerned about the possibility that research implemented by international organizations could pose a political threat through the ‘exposure of information’ about how people who use illicit drugs are treated in the CCT centers. Because of this concern, the new DOLISA leader requested the research team submit the study protocol, the questionnaires and all related recruitment materials for his review and endorsement, in consultation with other relevant government departments (the Department of Police, the People’s Committee, the Department of External Affairs).

Navigation between science and politics was required for the study to move forward. Similar to the randomized controlled trial of heroin-assisted treatment in Canada conducted in 2009 [45], political considerations influenced study design to some degree. For this study conclusions have been drawn using proxy data. In addition, interviews of participants were only allowed in one location to enable monitoring of the research activities by the local government. Without this constraint, interviews could also have been conducted in the neighborhoods near the homes of study participants. This may have further increased the follow-up rates (notwithstanding the high rates achieved: 80% for CCT participants), hence increasing the level of confidence in the study conclusions. Last but not least, the need for a renewed political approval following a change in the leadership of Hai Phong City DOLISA came at the cost of a substantial delay for the study. This caused variations between the original design and the actual recruitment and participant interview timelines.

As mentioned previously, the ultimate goal of conducting this research was to generate evidence to support dialogue for drug policy change in Vietnam. The main results of the cost-effectiveness analysis demonstrated that MMT is not only more effective but also less expensive than CCT in achieving additional drug-free days [26]. The conclusiveness of the study findings was critical for gaining acceptance from the political audience who were the targeted end-users of this study. In April 2015, three meetings were organized by FHI360 to disseminate the results of the study. The first meeting was for all the relevant government agencies in Hai Phong City. The second meeting was for the international agencies who work on drug dependence treatment and HIV in Vietnam and the third meeting was for the Advisory Board, whose members are retired ministers, and whom provide advice to the Deputy Prime Minister on drug policy, civil society organizations and the representatives of three key ministries (Ministry of Labor, Invalids and Social Affairs, Ministry of Health and Ministry of Public Security). In all three meetings, the audience was not surprised by the findings of the study. Despite some discussion of the limitations of the study (non-randomization and time-frame), the study findings were well-received. Anecdotally, for a long time, there had been a belief that MMT is likely to be less costly and more effective than CCT. But there had been no empirical data to substantiate this belief. Without the confirmation of empirical data, the work of policy advocacy (of both international agencies and government agencies) for evidence-informed policy-making had been extremely challenging. The process has resulted in the official publication and dissemination of the study findings report (in both English and Vietnamese). In July 2015, the Advisory Board took ownership of the study and presented the study findings to the Deputy Prime Minister. The outcome was a letter from the Chairman of the Office of the Government giving authorization to the Advisory Board to disseminate the findings of the study via a variety of communication media, including via official visits to the provinces and via the official website of the Office of the Government “Tieng Chuong” (The Bell) (ref Letter 6021/VPCP-KHVX dated 31 July 2015).

Nevertheless, it would be naïve to think that a single study could lead to significant change in drug policy-making. Rather, policy change occurs through the culmination of efforts and strategies, from a range of different actors across many years of advocacy work. If this study had never been conducted, positive change in Vietnam would still have occurred. However, the findings of this study assisted by facilitating more evidence based conversations between advocacy agencies and government decision-makers, hence speeding up the process of change. Politicians and government bureaucrats who used to be ambivalent might be able to reconsider their position. Members of the Advisory Board to the Deputy Prime Minister have taken ownership of the study findings in their conversations with leadership of local jurisdictions. For Vietnam, the road from policy to implementation will be long. Progress is likely to be challenged by existing laws and policies, the lack of skilled human resources and infrastructure to rapidly establish evidence-based community treatment in place of these CCT centers, the pervasive stigmatization of people who use drugs and the ongoing tension between the abstinence-based model of treatment as compared to harm reduction approaches.

### Lessons learned

We have learned much about the successes and challenges of conducting research around compulsory drug treatment in Vietnam. On the positive side, we have demonstrated that conducting research in this topic is feasible, largely due to the high levels of diligence and perseverance from the Vietnam research team leaders. But it was not only persistence and perseverance that was needed. The political endorsement was possible due to a combination of factors: the progressiveness of Hai Phong leadership (notwithstanding the delay in decision-making on granting final approval for the study), the local government’s trust in FHI360 (which implements the biggest US-funded HIV program in Vietnam), and FHI360’s 10-year financial and technical assistance to Hai Phong City for the implementation of a comprehensive HIV and drug dependence treatment program. Without any of the above factors, the conduct of this study may have been impossible.

Methodologically, some of the most important lessons concern the strategies for recruitment of participants and the independence of the research and clinical teams. An active recruitment carried out by well-trained local researchers and peer educators/outreach workers with good ties to the community that serves the target population was crucial to recruiting the needed number of CCT participants. Despite the marginalized nature of the study participants, the research team was able to follow-up 80% of the CCT participants and 78% of the MMT participants.

## Conclusions

Research on compulsory drug dependence treatment is feasible but politically challenging and requires navigation between science and politics. This paper shares lessons learned and offers assistance for the design and conduct of future research in this challenging topic in other Southeast Asian countries. The study findings have been and will continue to be used for drug policy advocacy in Vietnam [26]. Scientific evidence is only one type of information available to politicians. Information, including scientific and other types, has to compete with ideologies and self-interests to gain primacy in policy making. Evidence can make a difference, particularly in countries that take pragmatic approaches to health problems like Vietnam.

## Abbreviations

BBV: Blood-borne virus
CCT: Center-based compulsory treatment
HIV: Human Immunodeficiency Virus
INCB: International Narcotics Control Board
MMT: Methadone maintenance treatment
UNODC: The United Nations Office on Drugs and Crime
